# Effects of aerobic exercise on metabolic indicators and physical performance in adult NAFLD patients: A systematic review and network meta-analysis

**DOI:** 10.1097/MD.0000000000033147

**Published:** 2022-04-07

**Authors:** Yangjun Liu, Wei Xie, Juan Li, Zbigniew Ossowski

**Affiliations:** a School of Physical Education and Health, Chengdu University of Traditional Chinese Medicine, Wenjiang District, Chengdu, Sichuan Province, China; b Faculty of Physical Culture, Gdansk University of Physical Education and Sport, Poland, Gdańsk, Poland; c Graduate School of Chengdu Sport University, Chengdu, Sichuan, China.

**Keywords:** aerobic, exercise, metabolic diseases, physical performance, randomized controlled trials

## Abstract

nonalcoholic fatty liver disease (NAFLD) is characterized by excessive accumulation of fat in the liver without alcoholism. We conducted a systematic review and meta-analysis to elucidate the efficacy of aerobic exercise on metabolic indicators and physical performance of adult patients with NAFLD.

**Methods:**

To conduct the systematic review and network meta-analysis, 2 researchers searched PubMed, EBSCO, and Web of science databases to identify randomized clinical trials of aerobic exercise interventions for adults with NAFLD published between inception and July 2022. We assessed the methodological quality of the included literature using the Cochrane Risk Assessment Scale and the PEDro Scale. Relevant data were extracted, variables were converted to the same units, and meta-analysis was performed using RevMan 5.4 software. We compared mean differences (MD) between experimental and control groups. For each outcome analyzed, we expressed data as MD with 95% CI to compare metabolic markers and exercise capacity between the experimental and control NAFLD patients.

**Results:**

Eleven randomized clinical trials with a total of 491 individuals with NAFLD were included in accordance with the criteria of this study. Types of aerobic exercise include moderate or high-intensity interval running, cycling, Nordic walking, equipment training, etc; Training duration 4 to 16 weeks, 30 to 60 minutes 3 or more times a week. Compared with the control group, aerobic exercise group had reduced weight of patients, (MD) 1.20 kg (95% CI: −1.38 to −1.01 kg, *P* < .00001). Seven studies confirmed that aerobic exercise significantly reduced triglycerides, (MD) 3.00 mg/dL (95% CI: −5.80 to −0.21 mg/dL, *P* = .04); increased high density lipoproteins (MD) 5.96 mg/dL (95% CI: 2.95 to 8.96 mg/dL, *P* = .0001) and reduced low-density lipoproteins (MD) 6.45 mg/dL (95% CI: −8.53 to −4.37 mg/dL, *P* < .00001); the study also showed that aerobic exercise reduced the liver enzymes aspartate aminotransferase and alanine aminotransferase to varying degrees. Aerobic exercise can improve physical performance and increase peak oxygen consumption of (MD) 6.29 mL/Kg*minutes, (95% CI: 3.05–9.53mL/Kg*minutes, *P* = .0001).

**Conclusion:**

Aerobic exercise significantly reduced weight and improved metabolic index and physical performance. Impacted by the limitations of various regimens, doses, duration, center settings, populations enrolled, the study had certain limitations. The randomized controlled trials with larger sample sizes, multiple centers, and high quality should be conducted to validate the above conclusion. Further studies will be required to focus on the total duration of the intervention, duration and frequency of sessions, and intensity that are optimal for the promotion of physical performance and metabolic capacity in this population.

## 1. Introduction

nonalcoholic fatty liver disease (NAFLD) is very common, affecting approximately 1 billion people worldwide^[1]^ and is the leading cause of chronic liver disease in developing countries, with a prevalence of 20% to 35% in Western countries.^[2]^ In China, the prevalence of NAFLD is 10% to 30%, especially in obese and type II diabetes mellitus patients, with a prevalence of up to 75%.^[3]^ Currently, there are no drugs to treat NAFLD, and the recommended treatment options include weight loss through exercise and a balanced diet.^[4]^ Studies have shown that aerobic exercise as an economical and convenient intervention has a significant impact on both obesity management and NAFLD improvement.^[5]^

Different types of aerobic exercise, as well as the intensity and duration of exercise training, have different effects in individuals with NAFLD. In terms of the quality of the evidence, the conclusions of aerobic exercise intervention in NAFLD are not deep enough, which is not conducive to the development of evidence-based practice of aerobic exercise intervention in NAFLD disease.^[6–9]^ In this review, a systematic review and meta-analysis were conducted to explore whether aerobic exercise could bring more benefits to patients compared with conventional treatment, and to provide a relevant basis for the application of aerobic exercise in NAFLD rehabilitation.

## 2. Method

### 2.1. Protocol

We conducted systematic reviews and network meta-analyses following the Preferred Reporting Items for PRISMA Systematic Reviews and Meta-Analysis statement.

### 2.2. Search strategy

We conducted a systematic search of PubMed, EBSCO, and the Web of Science between May and July 2022. Articles published until July 2022 cover key concepts for nonalcoholic fatty liver, aerobic exercise, and adults or the elderly. All searches were limited to randomized clinical trials (RCTs) in humans, and no language limits were set (Supplemental Digital Content, http://links.lww.com/MD/I772). Moreover, the reference lists cited in relevant systematic reviews and included trials were screened. In addition, the 2 authors each manually searched the proceedings of major international conferences, systematic reviews, meta-analysis and gray literature to recursively search potential studies to prevent missing relevant studies for which only abstracts are available. Furthermore, all initial search results were screened by 2 blinded investigators independently.

### 2.3. Incorporation/exclusion criteria

Based on the inclusion and exclusion criteria, 2 researchers independently screened the literature, extracted the data, and cross-checked the data. If there was a disagreement, they discussed the resolution or consulted a third-party opinion. Literature screening began with the title and abstract, and after excluding clearly irrelevant literature, the full text was further read to determine whether it would eventually be included.

#### 2.3.1. Inclusion criteria

Inclusion criteria were based on the study population, interventions, comparative questions (controls), and outcomes.^[10]^

- Investigate the source of nonalcoholic liver disease (18 or older) (explicitly NAFLD) as confirmed by at least one of the following protocols: liver biopsy, ultrasound, computed tomography (CT), magnetic resonance imaging, serum bile acids, γ-glutamyl transpeptidase, aminotransferases, aspartate aminotransferase (AST), alanine aminotransferase (ALT), or bilirubin, alkaline phosphatase, and dyslipidemia.^[11–15]^
- Application of aerobic exercise as an intervention. Joint exercise was defined as an intervention that used aerobic training and other types of training simultaneously.
- Exercise alone or in combination with other interventions (education, nutrition, etc); there must be a control group (CG) and a group that only performs aerobic exercise (to determine its independent effect). The CG did not have any interventions (e.g., nutritional or physical interventions) that might have affected the measurement of the study results. In addition, the exercise group (EG) participants had to train at least 3 times a week for at least 4 weeks to determine the long-term effects of exercise on the variable. Studies must be conducted before and after the intervention to determine changes in the variables.
- A peer-reviewed study.
- Written in English only.

#### 2.3.2. Exclusion criteria

- Bibliographic reviews, uncontrolled or randomized clinical trials, and animal models.
- Participants had comorbidities, such as type II diabetes, viral hepatitis, cardiovascular disease, lung disease, or neurological disease.

Other interventional interventions.

### 2.4. Data extraction

After reading the full article, the literature was imported to Microsoft Excel. One researcher extracted the data and 2 researchers reviewed it to ensure the accuracy of data processing (see Table 1). The data included the following parameters: the last name of the first author’s year of publication, number of participants in the group, type, intensity, frequency, and duration of exercise interventions, and methodology for evaluating NAFLD impact indicators. We only extracted the data that matched the study and displayed the results of the synthesis in the Data Synthesis section.

**Table 1:** Included studies – exercise programming details and evaluation indicators.

	n		Aerobic training			Indicator	
study	EG	CG	Duration	Intensity	Method	Physiological	Exercise capacity
Ghamarchehreh et al, 2019	13	13	45 min 3 x/wk 8 wk	55–75% HRR	Running	TG Chol. HDL LDL	NA
Hoseini et al,2020	10	10	45–60 min 3 x/wk 8 wk	60–75% MHR	Running or walking	BW BMI BPF WHR TG TC LDL HDL Glucose Insulin HOMA-IR Vitamin D	NA
Whyte et al,2020	15	12	20–60 min 4–5 x/wk 16 wk	40–60% HRR	Gym-based or other modes of exercise	BW BMI HOMA2 S ALT VLDL LDL HDL-C IHCL	VO_2max_
Pugh et al,2014	13	8	30–45 min 3–5 x/wk 16 wk	30–60% HRR	Running and other gym aerobics	Waist Glucose SAT FMD	VO2, V O2 peak,
Abdelbasset et al,2020	15	16	40–50 min 3 x/wk 8 wk	60–70% HRR	Cycling ergometer (Monarkrc6, Novo Langley, USA)	TG LDL TV Cl VF HbA1C DL AL HOMA-IR BMI	
Cheng et al,2017	29	29	30–60 min 2–3 x/wk 36 wk	60–75% VO_2max_	Nordic brisk walking + stretching and other group exercises	HFC	
Abdelbasset et al,2019	16	16	40 min 3 x/wk 8wk	50–85% of the VO_2max_	Cycling	IHTG IHCL HbA1C glucose HRQoL	VO_2peak_
Zhang et al,2016	146	74	>30 min 5 x/wk 1 yr	65–80% HRR	Running/walking	IHTG BW Fat	
Winn et al,2017	16	5	60 min 4 x/wk 4 wk	MICT/HIIT	Exercise training and aerobic fitness	IHL VF HDL LDL	VO_2peak_
Pugh et al,2013	6	5	30–45 min 3–5 x/wk 16 wk	30–60% HRR	Running and other gym aerobics	VAT SAT ALT AST GGT Glucose TG TC HDL LDL	VO_2peak_
Houghton et al,2013	12	12	30–45 min 3 x/wk 12 wk	60–70% HRR	Cycling and resistance exercise	HTGC VF TG	

AL = alanine transaminase, AST = aspartate aminotransferase, ALT = alanine aminotransferase , BMI = body mass index, BPF = body percent fat, CG = control group, DL = density lipoproteins, EG = exercise group, GGT = γ-glutamyl transpeptidase, FMD = flow mediated dilatation, HbA1C = glycated hemoglobin, HDL = high density lipoproteins, HFC = hepatic fat content, HIIT = high-intensity interval exercise training, HOMA-IR = homeostatic model assessment-insulin resistance, HRQoL = health-related quality of life, HRR = heart rate reserve, IHCL = intrahepatic lipids, IHTG = intrahepatic triglyceride, LDL = low-density lipoproteins, MHR = maximum heart rate, MICT = moderate-intensity continuous exercise training, NA = not available, S = homeostasis model assessment, SAT = subcutaneous adipose tissue, HOMA2, TC = total cholesterol, TG = triglycerides, VF = visceral fat, VLDL = very-low-density lipoprotein, VO2max = maximal oxygen consumption, VO_2peak_ = peak oxygen consumption, WHR = waist-to-hip ratio, wk = week.

### 2.5. Risk of bias and literature quality evaluation

We assessed the methodological quality of the included studies using the Cochrane Risk Assessment Scale and the PEDro Scale. The risk of bias assessment included selection bias (random sequence and allocation concealment), performance bias (blinding of participants and researchers), detection bias (blinding of outcome assessment), attrition bias (incomplete outcome data), reporting bias (selective reporting), and other sources of bias. Two review authors independently scored the articles, and discussions were held with a third review author when the results were inconsistent.

### 2.6. Outcome measures

For the RCTs, all reported outcome indicators relating to metabolic indicators and physical performance were evaluated, with the primary outcome of (I) total cholesterol, triglycerides (TG), high density lipoproteins (HDL) and low-density lipoproteins (LDL); and the secondary outcome of (II) alanine transaminase.

T, AST, peak oxygen consumption (VO_2peak_) and body weight (BW). The above have been extensively used in NAFLD patients.

### 2.7. Data synthesis

When 3 or more included studies reported the same outcomes, we performed a meta-analysis using pooled data from RevMan 5.4 according to the Cochrane Manual for Systematic Review of Interventions. The mean, standard deviation, and sample size of the extracted data were entered into the statistical software. The extracted variables were converted to the same units and the mean differences between the intervention and CGs were compared. For each outcome analyzed, we represented the data as the mean differences with 95% CI. Standardized mean difference was used to express the effect size.

### 2.8. Heterogeneity

In all analyses, *I*^2^ statistics were used to analyze heterogeneity between studies. Higher *I*^2^ values indicate greater heterogeneity.^[16]^ If the heterogeneity test value was *P* < .05 (*I*^2^ statistic), a random-effects model was used; otherwise, fixed-effects models were used to account for differences in study participants and the amount and intensity of the exercise interventions.

## 3. Results

### 3.1. Studies selection

**Figure 1.**
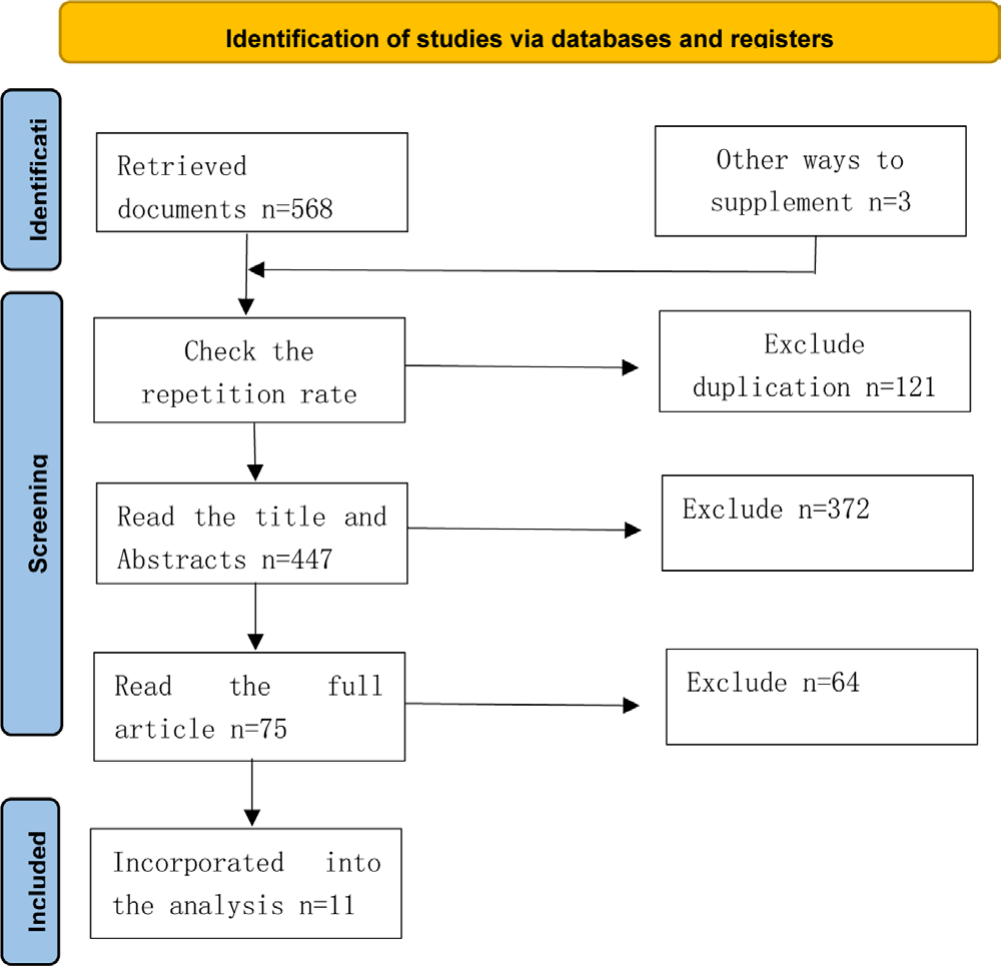
Flow diagram of the study identification and selection process.

We identified 568 articles in PubMed, EBSCO, and Web of Science, and added 3 more articles through a manual search. A total of 121 duplicate articles were excluded and 372 were excluded after analyzing the titles and abstracts. Of the remaining 75 articles, we carefully read the full text and excluded 64. The main reasons for exclusion were nonconformity of intervention mode, combination intervention, drug intervention, and outcome. We included 11 articles in the final analysis (Fig. 1).

### 3.2. Description of the included studies

In total, 491 participants were included in the analysis. Of these, 291 participated in the exercise intervention and 200 participated in the CG. Three studies used moderate-intensity aerobic training regimens,^[17–19]^ 7 studies used moderate-intensity and high-intensity aerobic training regimens,^[20–26]^ and 1 study used high-intensity intermittent aerobic training regimens.^[27]^ Eleven studies involved exercise intervention. All studies had a CG consisting of patients with NAFLD who received standard or routine care and did not include exercise interventions or any other factors that may have affected the outcome. The duration of the exercise intervention was 4 to 16 weeks, in which the training frequency was 3 to 5 times a week for 20 to 60 minutes each time. Moderate training intensity is 30% to 60% of the heart rate reserve (HRR) medium to high training intensity is 60% to 75% of HRR and High Training Intensity Intermittent Training HRR ranges from 50% to 85%. Aerobic training methods included bicycles, treadmills, Nordic brisk walks, walking, and optional gym exercises.

### 3.3. Quality assessment analysis

Risk of bias assessment was performed according to the Cochrane Risk of Bias Assessment Scale. All 11 included studies reported on participants baselines, were parallel and comparable, mentioned randomized controlled trials in the literature, and 11 described specific randomization methods, such as random number tables and computer randomization; Ten were allocated concealed and at low risk of bias. All the participants signed an informed consent form. Quality evaluation was performed using the PEDro scale^[28]^ (see Table 2). Due to the nature of exercise interventions, almost all articles were not immune to performance bias. Specific blinding of the outcome assessment was not reported in ten studies. Eight studies described the reasons for loss to follow-up, one of which used intention-to-treat.^[23]^

**Table 2:** PEDro scale scores of the included studies.

Study\score	1	2	3	4	5	6	7	8	9	10	11	Total	Quality
Ghamarchehreh et al, 2019	Y	Y	Y	Y	N	N	N	Y	N	Y	Y	6	M
Hoseini et al, 2020	Y	Y	Y	Y	Y	N	N	Y	N	Y	Y	7	H
Whyte et al, 2020	Y	Y	Y	Y	N	N	N	Y	N	Y	Y	6	M
Pugh et al, 2014	Y	Y	Y	Y	N	N	N	Y	N	Y	Y	6	M
Abdelbasset et al, 2020	Y	Y	Y	Y	Y	N	N	Y	N	Y	Y	7	H
Cheng et al, 2017	Y	Y	Y	Y	N	Y	Y	Y	Y	Y	Y	8	H
Abdelbasset et al, 2019	Y	Y	Y	Y	N	N	N	Y	N	Y	Y	6	M
Zhang et al, 2016	Y	Y	Y	Y	N	Y	N	Y	N	Y	Y	7	H
Winn et al, 2017	Y	Y	N	Y	N	N	N	Y	N	Y	Y	5	M
Pugh et al, 2013	Y	Y	Y	Y	N	N	N	Y	N	Y	Y	6	M
Houghton et al, 2013	Y	Y	Y	Y	N	N	N	Y	N	Y	Y	6	M

1 = eligibility criteria and source, 2 = random allocation, 3 = concealed allocation, 4 = baseline comparability, 5 = participants, 6 = blinding of therapists, 7 = blinding of assessors, 8 = adequate follow-up (85%), 9 = intention-to-treat analysis, 10 = intergroup statistical comparisons, and 11 = reporting of point measures and measures of variability.

### 3.4. Results of the meta-analysis

#### 3.4.1. Effects of aerobic exercise on BW and visceral fat (VF)

**Figure 2.**
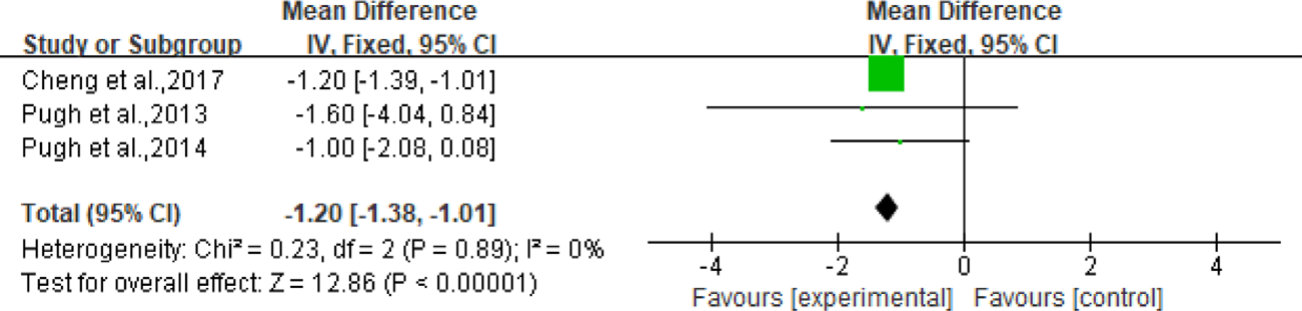
Forest plot for body weight (kg).

The effects of aerobic exercise on BW were reported in 11 studies. Ghamarchehreh et al^[20]^ reported that aerobic exercise had no significant effect on weight reduction, and Houghton et al^[26]^ required weight preservation throughout the process; therefore, there was no change in weight before and after the experiment. Aerobic exercise causes significant weight loss and body mass index (BMI).^[17–19,21–25,27]^ Three^[18,19,23]^ studies reported an average reduction of 1.20 kg of BW (95% CI: −1.38 to −1.01 kg, *P* < .00001) with aerobic exercise, see Figure 2. Weight loss was also observed in the absence of dietary intervention and with 150 minutes or less of weekly exercise. Almost all 11 selected studies measured liver fat. These findings suggest that aerobic exercise without dietary changes can also be effective in reducing liver fat.^[23,25,27]^

#### 3.4.2. Effect of aerobic exercise on the patient’s metabolic parameters

**Figure 3.**
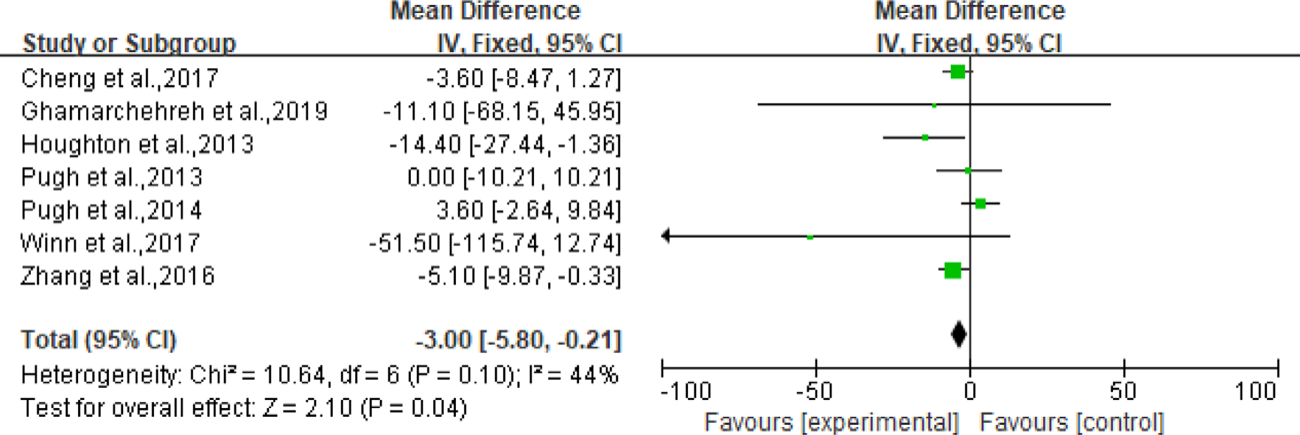
Forest plot for TG (mg/dL). TG = triglycerides.

**Figure 4.**
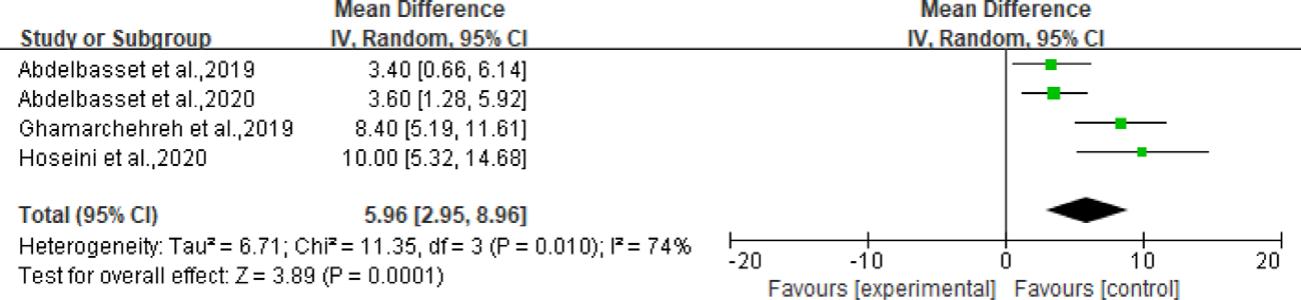
Forest plot for HDL (mg/dL). HDL = high density lipoproteins.

**Figure 5.**
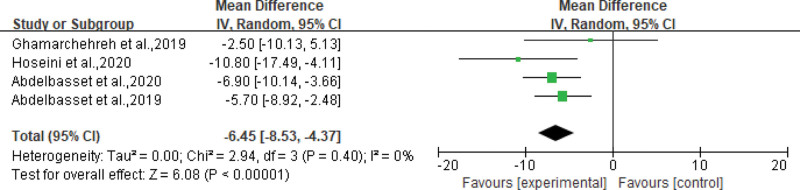
Forest plot for LDL (mg/dL). LDL = low-density lipoproteins.

All the included studies reported the effects of aerobic exercise on metabolic parameters in the experimental group. Most studies have used TG, HDL, LDL, ALT, and AST as indicators to reflect the effect of aerobic exercise on metabolic parameters in patients with NAFLD. Seven^[18–20,23–26]^ studies demonstrated that aerobic exercise significantly reduced blood TG levels, with an average reduction of 3.00 mg/dL (95% CI: −5.80 to −0.21 mg/dL, *P* = .04), see Figure 3. Four^[20–22,27]^ evaluated the effects of aerobic exercise on HDL and LDL levels. We performed a meta-analysis of the results obtained after exercise, comparing the changes in HDL and LDL levels in the CG with those in the aerobic EG. Among them, aerobic exercise increased HDL by an average of 5.96 mg/dL (95% CI: 2.95 to 8.96 mg/dL, *P* = .0001) in patients with NAFLD, see Figure 4. At the same time, LDL was reduced by an average of 6.45 mg/dL (95% CI: −8.53 to −4.37 mg/dL, *P* < .00001), as shown in Figure 5. In addition, 6^[17,21,22,25–27]^ studies showed that aerobic exercise led to varying degrees of reduction in liver enzymes AST, ALT, and cholesterol.

#### 3.4.3. Effects on exercise ability

**Figure 6.**
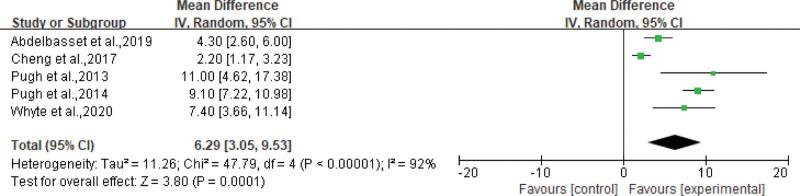
Forest plot for VO_2peak_ (mL/Kg*min). VO2peak = peak oxygen consumption.

We conducted a meta-analysis of the results obtained from the change in exercise ability of the participants after exercise, comparing the changes in physical ability in the CG with those in the aerobic EG. Five^[17–19,23,27]^ studies provided data on changes in physical capacity. The maximum oxygen consumption (VO_2max_) or VO_2peak_ of the subjects was determined according to the BPET (cardiopulmonary exercise experiment) and expressed in mL/kg*minutes. Five studies reported a significant increase in the VO_2max_ or VO_2peak_. The pooled analysis of the study showed that aerobic exercise increased VO_2peak_ by an average of 6.29 mL/Kg*minutes, (95% CI: 3.05–9.53 mL/Kg*minutes, *P* = .0001), see Figure 6.

## 4. Discussion

nonalcoholic fatty liver disease, characterized by steatosis (lipid storage in the liver > 5.5%), is an important factor in the development of type II diabetes and cardiovascular disease. Its main manifestations of NAFLD include obesity, high VF content, changes in metabolic parameters such as high TG, HDL, LDL, ALT, and AST, and CRF are strongly associated with changes in liver fat and are useful indicators for determining changes in liver fat caused by exercise interventions.^[29,30]^ Insufficient exercise capacity is also an important reason for further development of NAFLD-related indicators. The health intervention status of aerobic exercise in NAFLD patients can be effectively explored by evaluating the effect of aerobic exercise on the exercise ability of these patients. We discuss the effects of aerobic exercise on patients with NAFLD in 3 aspects: weight change, metabolic indices, and improvement of exercise capacity.

### 4.1. Effects on BW and liver fat

Although the amount, intensity, frequency, and mode of exercise varied, the vast majority of selected studies reported the effect of aerobic exercise on the weight of people with NAFLD. Five studies showed a significant effect of aerobic exercise on BW, 3 of these studies with a pooled mean reduction of 1.20 kg (95% CI: −1.38 to −1.01 kg). Taking 16 weeks of moderate-intensity aerobic exercise with an HRR of 3060 percent as an example, the average weight of the trainees can be reduced by 3.6 percent, and the BMI is also significantly reduced, which will be beneficial for the improvement of the health of patients with NAFLD.^[17]^ Although studies have shown differences in exercise intensity, duration, and training style, aerobic exercise has been shown to reduce BW and BMI^[22,25,27]^ in patients with NAFLD.^[18,19,21,23,24]^ Patients with overweight NAFLD exhibit chronic endothelial dysfunction and are more likely to have CVD risk,^[18]^ which is the leading cause of death in patients with NAFLD^,[31]^ and improving BW is important for cardiovascular health in patients with NAFLD. Studies have shown that 30 to 60 minutes of moderate-intensity aerobic exercise 3 times a week for more than 4 weeks can have a significant effect on weight reduction in patients with NAFLD. Although the selected studies have certain limitations, in future research and practice, aerobic exercise still could be used as an effective method for weight control in patients with NAFLD.

Several studies have reported that weight loss is accompanied by a decrease in liver fat. Whyte’s study noted that subjects liver fat levels decreased from 19.6% before training to 8.9% after training.^[17]^ Most of the aerobic exercise regimens in this review last 30 to 60 minutes/time, and some even had lower aerobic activity than the WHO recommended 150 minutes of aerobic exercise per week but still had a significant impact on reducing liver fat. Three^[23,25,27]^ studies showed that aerobic exercise had a significant effect on liver lipid content in the liver and that aerobic exercise could also be effective in reducing liver fat without dietary changes.^[21]^ However, the results of 1^[18]^ study showed a 3.3% decrease in liver fat percentage between exercise training and routine care 30% to 60% 3 to 5 times per week for a duration of 30% to 60%, but did not show a statistically significant difference (95% CI, −10.0 to 3.4, *P* = .18). This result may be related to the small sample size and duration of exercise. Studies have demonstrated that aerobic exercise can reduce abdominal and VF without causing weight loss.^[32–34]^ Therefore, future studies should explore the evidence for aerobic exercise and liver fat loss using larger samples or different intensities and durations.

### 4.2. Effect on metabolic indicators

NAFLD is considered a hepatic manifestation of metabolic syndrome and coexists with a variety of cardiometabolic risk factors including obesity, insulin resistance, hypertension, and dyslipidemia.^[35]^ NAFLD is typically characterized by hepatic TG accumulation (5% by weight) and elevated levels of liver enzymes, including ALT and AST, and consumption occurs without excess alcohol (up to 20 g/day).^[29,30]^ According to scientific reports published by the American Heart Association, dyslipidemia: TG ≥ 1.7 mmol/L (150 mg/dL), HDL-C ≤ 1.0 mmol/L (40 mg/dL) (males), and ≤ 1.3 mmol/L (50 mg/dL) (women) increase the risk of fatty liver.^[36]^ Therefore, it is important to explore the effects of aerobic exercise on triglycerides. Studies have shown that aerobic exercise lasting ≥ 4 weeks at different intensities is useful for reducing triglycerides. Zhang Jun et al^[24]^ showed that strenuous exercise and moderate-intensity exercise were equally effective in lowering intrahepatic triglycerides and inferred that the decline in triglycerides was mainly caused by weight loss. Houghton et al^[26]^ had shown that 12 weeks of aerobic exercise could lead to a 23% decrease in circulating triglyceride levels.

Aerobic exercise has a significant effect on HDL and LDL, which reduces plasma LDL and, on the other hand, raises plasma HDL. In the aerobic group, the decrease in LDL showed a natural correlation, as we observed a decrease in the percentage of body fat, as well as a decrease in liver fat. Multiple studies have shown that aerobic exercise reduces total cholesterol and LDL levels in patients with NAFLD and increases plasma HDL.^[20–22,27]^ Multiple studies have found a significant decrease in plasma AST and ALT levels in the aerobic EG and have used this as a clinical basis for recommending moderate-intensity aerobic exercise in patients with NAFLD.

### 4.3. Effects of physical performance

Individuals with NAFLD often suffer from obesity and lack of exercise, which leads to reduced exercise capacity. Oxygen consumption is one of the criteria for physical performance, and patients with NAFLD are assessed using the CPET method, which is a globally used comprehensive assessment of physiological response.^[37,38]^ Five of the selected studies used CPET to assess the comprehensive physiological response of patients with NAFLD, and the study reflected subjects exercise ability using VO_2max_ or VO_2peak_ as the maximum aerobic capacity indicator. The aerobic endurance training parameters applied in these studies are consistent with the European Association’s clinical practice guidelines for the management of NAFLD to study liver, diabetes and obesity; They describe an integrated approach to treating these patients.^[38]^ Our meta-analysis showed that after aerobic training, the athletic performance of the body improved. The overall analysis showed that physical performance improved significantly, regardless of their characteristics (type, intensity, and duration). Suchomel et al^[39]^ also showed that supervised exercise training improves VO_2peak_ by 9.1 mL/Kg*minutes compared with traditional care, which reportedly reduces the risk of all-cause mortality and cardiovascular events by 34% and 39%, respectively.

Therefore, our meta-analysis showed that aerobic exercise improved physical performance in patients with NAFLD. Follow-up after the study revealed that regular exercise in these patients maintained its beneficial effects. Therefore, aerobic exercise is a therapeutic strategy to improve cardiopulmonary health in patients with fatty liver disease.

### 4.4. Limitations

The main limitation of the studies was the small number of RCTs found, the studies selected by the Institute were the difficulty of ensuring that participants, therapists, and evaluators were blinded and that participants fully complied with the requirements of the training protocol. It is very difficult for the selected study to achieve double-blind results for participants and researchers because patients and therapists need to know each other and need guidance from therapists. Assessor blinding is critical for reducing the risk of bias and needs to be clearly stated in RCT reports. Regarding noncompliance, given that attribution is a common feature of exercise RCTs, researchers should include the intent to handle the analysis in a prespecified manner. Another limitation was whether the CG exercised physical exercise, which is difficult to assess and will have an impact on low- to medium-intensity training, which hindered the conclusions of the analysis. These limitations should be considered in future RCTs.

In our meta-analysis, some randomized controlled trials had small sample sizes; therefore, their findings should be interpreted with caution. The sample size should be increased in future randomized controlled trials. There were significant differences in the exercise intensity, total duration, training frequency, and modality of the exercise training programs. The results also varied for different aerobic exercise intensities. Therefore, we recommend that aerobic exercise training for patients with NAFLD should be selected at the appropriate intensity according to the patient’s age and exercise ability.

All RCT participants lacked the classification of NAFLD severity, which was another limitation of the study. This classification is essential for the development of refined sports training programs and indicators of exercise intervention. Future RCTs should include this classification when planning and executing exercise regimens. Finally, a limitation of this study was the small number of randomized controlled trials found in the meta-analysis, which hindered the conclusions of the analysis.

## 5. Conclusion

This systematic review and meta-analysis provide evidence that aerobic exercise is an important means of improving exercise capacity and metabolic indicators in patients with NAFLD. A key new finding in this review is that aerobic exercise lasting more than 4 weeks reduces weight and liver fat. There was a significant correlation between changes in liver fat and commonly used clinical indicators, such as BW and liver metabolites. Aerobic exercise of moderate-intensity and above significantly improves the maximum aerobic capacity in patients with NAFLD, but evaluation of other exercise abilities is rarely covered. Therefore, future studies should consider aerobic training for other exercise abilities in patients with NAFLD to evaluate its effect in this case.

## Author contributions

**Conceptualization:** Zbigniew Ossowski.

**Data curation:** Yangjun Liu, Juan Li.

**Formal analysis:** Juan Li.

**Methodology:** Yangjun Liu, Wei Xie, Juan Li, Zbigniew Ossowski.

**Resources:** Yangjun Liu, Juan Li.

**Software:** Yangjun Liu, Juan Li.

**Supervision:** Zbigniew Ossowski.

**Visualization:** Zbigniew Ossowski.

**Writing – original draft:** Yangjun Liu.

**Writing – review & editing:** Wei Xie, Zbigniew Ossowski.

## Supplementary Material

## References

[1] Loomba R Sanyal AJ. The global NAFLD epidemic. Nat Rev Gastroenterol Hepatol. 2013;10:686–90. 24042449 10.1038/nrgastro.2013.171

[2] Angulo P. GI epidemiology: non-alcoholic fatty liver disease. Aliment Pharmocol Ther. 2007;25:883–9. 10.1111/j.1365-2036.2007.03246.x 17402991

[3] Fan JG. Epidemiology of alcoholic and non-alcoholic fatty liver disease in China. J Gastroenterol Hepatol. 2013;28(Suppl 1):11–7. 23855290 10.1111/jgh.12036

[4] Chen HT Huang HL Li YQ. Therapeutic advances in non-alcoholic fatty liver disease: a microbiota-centered view. World J Gastroenterol. 2020;26:1901–11. 32390701 10.3748/wjg.v26.i16.1901 PMC7201149

[5] Slentz CA Bateman LA Willis LH. Effects of aerobic vs. resistance training on visceral and liver fat stores, liver enzymes, and insulin resistance by HOMA in overweight adults from STRRIDE AT/RT. Am J Physiol Endocrinol Metab. 2011;301:E1033–9. 21846904 10.1152/ajpendo.00291.2011 PMC3214001

[6] Li Y He F He Y. Dose–response association between physical activity and non-alcoholic fatty liver disease: a case–control study in a Chinese population. BMJ Open. 2019;9:e026854. 10.1136/bmjopen-2018-026854 PMC6475196 30928957

[7] Asada F Nomura T Hosui A. Influence of increased physical activity without body weight loss on hepatic inflammation in patients with non-alcoholic fatty liver disease. Environ Health Preventative Med. 2020;25:18. 10.1186/s12199-020-00857-6 PMC7285793 32522147

[8] Kistler KD Brunt EM Clark JM. Physical activity recommendations, exercise intensity, and histological severity of non-alcoholic fatty liver disease. Am J Gastroenterol. 2011;106:460–8; quiz 469. 21206486 10.1038/ajg.2010.488 PMC3070294

[9] Mascaró CM Bouzas C Montemayor S. Association between physical activity and non-alcoholic fatty liver disease in adults with metabolic syndrome: the FLIPAN study. Nutrients. 2022;14:1063. 35268038 10.3390/nu14051063 PMC8912862

[10] Guyatt GH Oxman AD Kunz R. GRADE guidelines: 2. Framing the question and deciding on important outcomes. J Clin Epidemiol. 2011;64:395–400. 21194891 10.1016/j.jclinepi.2010.09.012

[11] Younossi ZM. Nonalcoholic fatty liver disease and nonalcoholic steatohepatitis: implications for liver transplantation. Liver Transpl. 2018;24:166–70. 29272073 10.1002/lt.25003

[12] Perumpail BJ Khan MA Yoo ER. Clinical epidemiology and disease burden of nonalcoholic fatty liver disease. World J Gastroenterol. 2017;23:8263–76. 29307986 10.3748/wjg.v23.i47.8263 PMC5743497

[13] van Deursen VM Damman K Hillege HL. Abnormal liver function in relation to hemodynamic profile in heart failure patients. J Card Fail. 2010;16:84–90. 20123323 10.1016/j.cardfail.2009.08.002

[14] Kong LZ Chandimali N Han YH. Pathogenesis, early diagnosis, and therapeutic management of alcoholic liver disease. Int J Mol Sci . 2019;20:2712. 31159489 10.3390/ijms20112712 PMC6600448

[15] Engelking LR Dasher CA Hirschowitz BI. Withinday fluctuations in serum bile-acid concentrations among normal control subjects and patients with hepatic disease. Am J Clin Pathol. 1980;73:196–201. 7188830 10.1093/ajcp/73.2.196

[16] Higgins JP Thompson SG. Quantifying heterogeneity in a meta-analysis. Stat Med. 2002;21:1539–58. 12111919 10.1002/sim.1186

[17] Whyte MB Shojaee-Moradie F Sharaf SE. HDL-apoA-I kinetics in response to 16 wk of exercise training in men with nonalcoholic fatty liver disease. Am J Physiol Endocrinol Metab. 2020;318:E839–47. 32286882 10.1152/ajpendo.00019.2020

[18] Pugh CJ Sprung VS Kemp GJ. Exercise training reverses endothelial dysfunction in nonalcoholic fatty liver disease. Am J Physiol Heart Circ Physiol. 2014;307:H1298–306. 25193471 10.1152/ajpheart.00306.2014

[19] Pugh CJ Cuthbertson DJ Sprung VS. Exercise training improves cutaneous microvascular function in nonalcoholic fatty liver disease. Am J Physiol Endocrinol Metab. 2013;305:E50–8. 23651847 10.1152/ajpendo.00055.2013

[20] Ghamarchehreh ME Shamsoddini AR Alavian SM. Investigating the impact of eight weeks of aerobic and resistance training on blood lipid profile in elderly with non-alcoholic fatty liver disease: a randomized clinical trial. Gastroenterol Hepatol Bed Bench. 2019;12:190–6. 31528301 PMC6668763

[21] Hoseini Z Behpour N Hoseini R. Co-treatment with vitamin D supplementation and aerobic training in elderly women with Vit D deficiency and NAFLD: a single-blind controlled trial. Hepat Mon. 2020;20:e96437.

[22] Abdelbasset WK Elsayed SH Nambi G. Effect of moderate-intensity aerobic exercise on hepatic fat content and visceral lipids in hepatic patients with diabesity: a single-blinded randomised controlled trial. Evid Based Complement Alternat Med. 2020;2020:1923575. 32351591 10.1155/2020/1923575 PMC7171632

[23] Cheng S Ge J Zhao C. Effect of aerobic exercise and diet on liver fat in pre-diabetic patients with non-alcoholic-fatty-liverdisease: a randomized controlled trial. Sci Rep. 2017;7:15952. 29162875 10.1038/s41598-017-16159-x PMC5698376

[24] Zhang HJ He J Pan LL. Effects of moderate and vigorous exercise on nonalcoholic fatty liver disease a randomized clinical trial. JAMA Intern Med. 2016;176:1074–82. 27379904 10.1001/jamainternmed.2016.3202

[25] Winn NC Liu Y Rector RS. Energy-matched moderate and high intensity exercise training improves nonalcoholic fatty liver disease risk independent of changes in body mass or abdominal adiposity – a randomized trial. Metab Clin Exp. 2018;78:128–40. 28941598 10.1016/j.metabol.2017.08.012

[26] Houghton D Thoma C Hallsworth K. Exercise reduces liver lipids and visceral adiposity in patients with nonalcoholic steatohepatitis in a randomized controlled trial. Clin Gastroenterol Hepatol. 2017;15:96–102.e3. 27521509 10.1016/j.cgh.2016.07.031 PMC5196006

[27] Abdelbasset WK Tantawy SA Kamel DM. A randomized controlled trial on the effectiveness of 8-week high-intensity interval exercise on intrahepatic triglycerides, visceral lipids, and health-related quality of life in diabetic obese patients with nonalcoholic fatty liver disease. Medicine (Baltimore). 2019;98:e14918. 30896648 10.1097/MD.0000000000014918 PMC6708753

[28] Cashin AG McAuley JH. Clinimetrics: physiotherapy evidence database (PEDro) scale. J Physiother. 2020;66:59. 31521549 10.1016/j.jphys.2019.08.005

[29] Targher G Day CP Bonora E. Risk of cardiovascular disease in patients with nonalcoholic fatty liver disease. N Engl J Med. 2010;363:1341–50. 20879883 10.1056/NEJMra0912063

[30] St George A Bauman A Johnston A. Independent effects of physical activity in patients with nonalcoholic fatty liver disease. Hepatology. 2009;50:68–76. 19444870 10.1002/hep.22940

[31] Ong JP Pitts A Younossi ZM. Increased overall mortality and liverrelated mortality in non-alcoholic fatty liver disease. J Hepatol. 2008;49:608–12. 18682312 10.1016/j.jhep.2008.06.018

[32] Golabi P Locklear CT Austin P. Effectiveness of exercise in hepatic fat mobilization in non-alcoholic fatty liver disease: systematic review. World J Gastroenterol. 2016;22:6318–27. 27468220 10.3748/wjg.v22.i27.6318 PMC4945989

[33] Orci LA Gariani K Oldani G. Exercise-based interventions for nonalcoholic fatty liver disease: a meta-analysis and meta-regression. Clin Gastroenterol Hepatol. 2016;14:1398–411. 27155553 10.1016/j.cgh.2016.04.036

[34] Benatti FB Lira FS Oyama LM. Strategies for reducing body fat mass: effects of liposuction and exercise on cardiovascular risk factors and adiposity. Diabetes Metab Syndr Obes. 2011;4:141–54. 21779146 10.2147/DMSO.S12143 PMC3138146

[35] Targher G Byrne CD. Clinical review: nonalcoholic fatty liver disease: a novel cardiometabolic risk factor for type 2 diabetes and its complications. J Clin Endocrinol Metab. 2013;98:483–95. 23293330 10.1210/jc.2012-3093

[36] Alberti KG Eckel RH Grundy SM. Harmonizing the metabolic syndrome: a joint interim statement of the international diabetes federation task force on epidemiology and prevention; national heart, lung, and blood institute; American Heart Association; world heart federation; international atherosclerosis society; and international association for the study of obesity. Circulation. 2009;120:1640–5. 19805654 10.1161/CIRCULATIONAHA.109.192644

[37] Milani RV Lavie CJ Mehra MR. Understanding the basics of cardiopulmonary exercise testing. Mayo Clin Proc. 2006;81:1603160311–1611. 10.4065/81.12.1603 17165639

[38] Weisman IM Weisman IM Marciniuk D. ATS/ACCP statement on cardiopulmonary exercise testing. Am J Respir Crit Care Med. 2003;167:211–77. 12524257 10.1164/rccm.167.2.211

[39] Suchomel TJ Nimphius S Stone MH. The importance of muscular strength in athletic performance. Sports Med. 2016;46:1419–49. 26838985 10.1007/s40279-016-0486-0

