# Supplementary material for: Effects of aerobic exercise on metabolic indicators and physical performance in adult NAFLD patients: A systematic review and network meta-analysis
Source: Medicine (Baltimore). 2022 Apr 7;102(14):e33147. doi: 10.1097/MD.0000000000033147 (PMC10082233; doi:10.1097/MD.0000000000033147)
Supplement: Supplementary file 1 [file medi-102-e33147-s001.pdf]

Supplemental Digital Content
Manuscript
Effects of aerobic exercise on metabolic indicators and physical performance in adult NAFLD patients: A Systematic review and Meta-analysis
The First Author
Yangjun Liu
Supplementary Method
Retrieval Strategy
1.1 Embase
#1″non-alcoholic steatosis″ OR ″non-alcoholic fatty liver disease″ OR ″fatty liver″ OR ″hepatic fat″ OR ″Liver fibrosis” OR “Liver fibrosis” OR ″Liver disease” OR “Fatty liver disease″ OR ″Obesity”
#2“aerobic exercise” OR “aerobic training” OR “physical activity” OR “exercise” OR “physical exercise”
#3“adult” OR “older adults” OR “elderly” OR “geriatrics” OR “aging” OR “senior” OR “seniors” OR “older people” OR “aged 65” OR “65+”
#1 AND #2 AND #3

1.2 PubMed
(″non-alcoholic steatosis″ OR ″non-alcoholic fatty liver disease″ OR ″fatty liver″ OR ″hepatic fat″ OR ″Liver fibrosis” OR “Liver fibrosis” OR ″Liver disease” OR “Fatty liver disease″ OR ″Obesity”) and (“aerobic exercise” OR “aerobic training” OR “physical activity” OR “exercise” OR “physical exercise”) and (“adult” OR “older adults” OR “elderly” OR “geriatrics” OR “aging” OR “senior” OR “seniors” OR “older people” OR “aged 65” OR “65+”).

1.3 Web of Science
((TS=(Non-alcoholic fatty liver disease, NAFLD, fatty liver)) AND TS=(aerobic exercise, aerobic training, physical activity, exercise, physical exercise)) AND TS=(adult or older adults or elderly or geriatrics or aging or senior or seniors or older people or aged 65 or 65+)
